# Current status of nano‐vaccinology in veterinary medicine science

**DOI:** 10.1002/vms3.1221

**Published:** 2023-07-24

**Authors:** Soheil Sadr, Parian Poorjafari Jafroodi, Mohammad Javad Haratizadeh, Zahra Ghasemi, Hassan Borji, Ashkan Hajjafari

**Affiliations:** ^1^ Department of Clinical Sciences Faculty of Veterinary Medicine Ferdowsi University of Mashhad Mashhad Iran; ^2^ Department of Chemistry Faculty of Science The University of Guilan Rasht Iran; ^3^ Department of Pathobiology Faculty of Veterinary Medicine Ferdowsi University of Mashhad Mashhad Iran; ^4^ Department of Pathobiology Faculty of Veterinary Medicine Islamic Azad University, Science and Research Branch Tehran Iran

**Keywords:** nanotechnology, nanovaccine, veterinary, zoonosis control

## Abstract

Vaccination programmes provide a safe, effective and cost‐efficient strategy for maintaining population health. In veterinary medicine, vaccination not only reduces disease within animal populations but also serves to enhance public health by targeting zoonoses. Nevertheless, for many pathogens, an effective vaccine remains elusive. Recently, nanovaccines have proved to be successful for various infectious and non‐infectious diseases of animals. These novel technologies, such as virus‐like particles, self‐assembling proteins, polymeric nanoparticles, liposomes and virosomes, offer great potential for solving many of the vaccine production challenges. Their benefits include low immunotoxicity, antigen stability, enhanced immunogenicity, flexibility sustained release and the ability to evoke both humoral and cellular immune responses. Nanovaccines are more efficient than traditional vaccines due to ease of control and plasticity in their physio‐chemical properties. They use a highly targeted immunological approach which can provide strong and long‐lasting immunity. This article reviews the currently available nanovaccine technology and considers its utility for both infectious diseases and non‐infectious diseases such as auto‐immunity and cancer. Future research opportunities and application challenges from bench to clinical usage are also discussed.

## Graphical abstract

Nanovaccines are successful because of the enhanced lowest immunotoxicity, antigen stability, better immunogenicity, the flexibility of the physical characteristics of nanomaterials and sustained release.

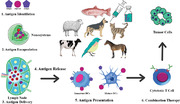

## 1 INTRODUCTION

Nanotechnology is a new field of science with various applications, including synthesizing different nanoparticle sizes and structures. In veterinary medicine, nanotechnology has enabled us to find treatments and develop powerful immune responses against infectious and non‐infectious diseases (Lei & Karim, 2021; Sahoo et al., 2021; Woldeamanuel et al., 2021). Nanotechnology finds its most notable application in cancer research, where it is primarily employed to enhance the detection and treatment of cancerous cells (Yang et al., 2021). According to recent studies (Alghuthaymi et al., 2021; Hu et al., 2020; Ross et al., 2019; Soliman et al., 2019), nanoparticles have emerged as a promising area of research for combating various types of diseases caused by microorganisms, such as fungi, bacteria and viruses. Nanoparticles possess unique properties that make them a potential tool for targeted drug delivery, diagnostic imaging and other therapeutic approaches. Nanoscience improves the imaging and diagnosis of diseases by removing biological restrictions (Tatli Seven et al., 2018). Various types of nanoparticles in veterinary medicine have been developed and characterized. Nanoparticles, such as liposomes, emulsions, proteasomes, nano‐beads, ISCOMs and polymeric biological nanoparticles like exosomes and bacteriophages, are being used to treat both non‐infectious and infectious diseases (Bai et al., 2018; Chariou et al., 2020; da Silva et al., 2021; Youssef et al., 2019). Nanoparticles are a powerful option for vaccine commerce due to the inactivity of surface change and the capacity to efficiently co‐transfer the adjuvants (Zaheer et al., 2021). Furthermore, the nano‐adjuvants in biological systems save the goal antigen from decay and improve absorption by immune intermediates (Sahu et al., 2018). This system leads to steady immunogenic effects and potentially delivers the antigen repetitively (Celis‐Giraldo et al., 2021). The size of nanoparticles significantly impacts their interaction with the immune system and how they are dispersed throughout the body. Nanoparticles that are smaller than 100 nanometres are readily taken up by dendritic cells (Shi et al., 2017). The nanoparticles can function as beneficial adjuvants, stimulating both cellular and humoral immune responses. As a result, the biomaterial can circulate more quickly, be more bioavailable, and have their biological components protected from degradation. In nanovaccines, regulating adjuvant and antigen size, surface charge, shape, hydrophobicity, flexibility and charge density is the key to improving a strong immune reaction by transferring nanovaccines to the lymphatic vessel. Polyethylene glycol (PEG) can slow down drug release from cells, which can increase the circulation time of the drug and help accumulate medication at the site of injury or damage (Prasad, Charmode, et al., 2021). Biodegradable nanovaccines can enhance slow release, antigen shelf life, immunogenicity and regular delivery to target cells (Cerbu et al., 2021). Nano‐medication is helpful in the prevention and treatment of diseases (Suzana Gonçalves Carvalho et al., 2020). Nanovaccines are far more efficient than conventional vaccines, and they can destroy infectious agents by activating the immune system in the blood and cells (Facciolà et al., 2019). Indeed, the stimulation of the immune responses can happen by transferring antigens to the immune system and providing the body with more powerful responses to the primary pathogen (Bhardwaj et al., 2020; Yadav et al., 2018). Nanoscience application in vaccine development has provided a novel method for solving the past questions. Nanovaccines also can deal with cancer treatment (Bragazzi, 2019; Ni et al., 2020). Tumour immunotherapy is a new medicinal way to inhibit tumour metastasis and recognize and kill cancer cells, by stimulating the patient's immune responses ( Asouli et al., 2023; Kheirollahpour et al., 2020; Prajapati et al., 2022). Within the field of immuno‐oncology, various modalities have emerged as promising approaches to enhance anti‐tumour immune responses. These include tumour vaccines, chimeric antigen receptor T‐Cell adoptive immunotherapy, and immune checkpoint inhibitors. These modalities have shown considerable efficacy in preclinical and clinical studies, offering new hope in the fight against cancer (Billingsley et al., 2020; Mi et al., 2019). In the past few years, cancer vaccines have proven to have hopeful therapeutic effects in scientific trials due to their convenient preparation, high specificity and relatively low cost (Cuzzubbo et al., 2021; El‐Sayed & Kamel, 2020). The main procedure of cancer vaccines is triggering and rapid increase of tumour antigen‐specific (CTL), cytotoxic T lymphocytes to destroy tumour tissues (Chen et al., 2021).

Nanoscience has gained significant attention in the field of veterinary medicine, with particular emphasis on its potential applications in diagnosis, vaccination and treatment. This article will provide a review of several Nano‐Biotech programs that are focused on advancing vaccination science in veterinary medicine.

## 2 TYPE OF NANOPARTICLES AND THEIR APPLICATIONS

A nanoparticle is a material with unique properties and a size range of 1–1000 nanometres. Nanoparticles with distinct chemical structures and physical properties could deliver molecules for their anthelmintic effects. They could be labelled as follows.

### 2.1 Polymeric structures

Generally, polymers are chain‐like structures with diverse characteristics. They can be natural polymers like polysaccharides, such as inulin, chitosan or synthetic, like PEG, other polyanhydrides, and polyesters (Han et al., 2018). Some synthetic polymers, like aliphatic polyesters, are environmentally friendly because of their decay characteristics. Moreover, hydrophobic and hydrophilic macromolecules can be encapsulated in polyester‐based polymers. Furthermore, natural biopolymers demonstrate benefits, such as low toxicity, biodegradability, abundance and biocompatibility (Davoodi et al., 2022). The most common polysaccharides in nano‐pharmacology are chitosan, dextran, agarose, hyaluronic acid and protein polymers, such as albumin, gelatin or soy (Cordeiro et al., 2015). Until now, chitosan has contributed to most studies, thanks to its non‐immunogenic impacts, great biocompatibility and the capacity to combine with other polymers. Due to these effects, chitosan is one of the most common molecules for cancer treatment (Chua et al., 2012). Polymeric nanoparticles include natural, artificial or semi‐synthetic polymers, so some challenges for preparing those structures will be the drug balance or compatibility and inert substance with the polymers (Guo et al., 2015). Several pure polymers can improve drug delivery systems, including pectin, carboxymethyl cellulose, alginate, hyaluronic acid, hypromellose phthalate, dextran and chitosan (Luo et al., 2017). The production technique performs a vital role in attaining the ideal properties of the nanoparticles, far from the natural features of polymers (Su et al., 2022). Polymeric nanoparticles have gained attention recently due to their potential applications in drug delivery, diagnostics and imaging. Various techniques have been developed for producing these nanoparticles, including reversed‐phase microemulsion, ionotropic gelation, emulsification solvent evaporation, emulsion polymerization, nanoprecipitation and supercritical fluid technology (George et al., 2019).

### 2.2 Lipid‐based nanoparticles

These nanoparticles are primarily created for lipophilic medication delivery and consist of a solid lipid matrix, surfactant and stabilizer. The lipid matrix components include glyceride mixtures, triglycerides and waxes (Thi et al., 2021). A further benefit of lipid nanoparticles and liposomes is that most of them are already authorized by European Medicines Agency and the Food and Drug Administration (García‐Pinel et al., 2019).

#### 2.2.1 Liposomes

Liposomes were presented in the 1970s as drug carriers, and today they are contained in some EU‐approved and USA‐formulated medications to decrease the harmful impacts of powerful medicines (Samimi et al., 2019). Liposomes are the most broadly defined nanoparticles in literature for veterinary applications. They are nanostructures shaped through double lipid layers composed of phospholipids (Ickenstein & Garidel, 2019). These nanoparticles offer numerous advantages, including their ability to interact with both water‐soluble and lipid‐soluble medications, their ability to be regulated by electric charge, their size range of 25–1000 nm and their capacity for functionalization (Kumar, 2019). Bangham & Horne (1964) defined these nanoparticles. Since then, numerous research with this nanodevice has been developed, displaying promise in multiple areas, like veterinary medicine. Nevertheless, several problems limit their use, like drug leakage, stability and upscaling (Ahmad et al., 2021).

#### 2.2.2 Solid lipid nanoparticles (SLN)

As an alternative to liposomes, solid lipid nanoparticles (SLN) appeared after 1990 (size levels 50–1000 nm). SLN may be controlled through all application methods; their manufacturing usually distributes natural liquids and is readily useful (Wang & Luo, 2019). Furthermore, biodegradable solid lipids can be utilized to form SLN, which is supported by surfactants to maintain stability. Therefore, the SLN nanoparticle technology is an innovative approach for creating NE (oil‐in‐water) formulations by replacing the liquid lipid with a solid form, resulting in even greater stability (Wang et al., 2021). Hence, it is possible to regulate the release and balance of the system under environmentally harmful situations. SLNs demonstrate several benefits, such as the capacity for parenteral routes, excellent stability, release control, specific targeting, and the physical safety of easily destroyable drugs (Geszke‐Moritz & Moritz, 2016). On the other hand, some problems stay, such as drug expulsion and limited medicine loading.

#### 2.2.3 Nanostructured lipid carriers (NLC)

In the mid‐2000s, nanostructured lipid carriers (NLCs) appeared as enhanced lipid‐based carriers to defeat the previous problems of SLN and liposomes. Nano lipid carriers account for the second‐generation SLN and the third‐generation NE (Tang et al., 2018). The biophysical stability of solid lipids prevents the deactivation of medication and overcomes issues related to its precursor, such as problems with drug transportation and storage. Similar to SLN, NLCs are particles with an aqueous external medium, which is stabilized with amphiphilic elements called surfactants in constructional solid lipids (Fernandes et al., 2021). However, a fragment of this solid lipid phase can be replaced by (oil) in the liquid phase, which prepares a formless structure, preventing its removal and promoting better drug encapsulation (Chuang et al., 2018). For nanotoxicology factors, the great biocompatibility of SLN and NLC was similar to that of Doktorovova et al. (2014).

### 2.3 Dendrimers

Dendrimers were broadly examined in drug delivery for anticancer and antimicrobials purposes, gene transfer, immunization and MRI imaging, but only a few methods were used for anthelmintic medicines (Tabatabaie et al., 2018). These molecules are hyper‐branched, containing an initiator centre, an internal layer of repetitive parts, and surrounding, multifunctional groups. The core both illustrates the molecules’ shape and encapsulates various types of particles (Hari et al., 2012). Each new layer or branch is named ‘generation’, and the second layer can transfer small particles. Furthermore, outer functional groups are connected to their surroundings. Their solubility, low cost, tuning ability and cell membrane make them appropriate for different use as drug carriers (Paleos et al., 2010). The first study for dendrimer was on polyamidoamine (PAMAM), adjusted by ethylenediamine or ammonia cores. The PAMAM functional characteristics and shape are highly similar to natural proteins, for example haemoglobin or insulin (Kheraldine et al., 2021). Recently, more dendrimer groups were introduced, for instance, tecto, polipropilenimine, multilingual, amphiphilic, chiral and micellar dendrimers (Sherje et al., 2018).

### 2.4 Metallic nanoparticles

Metallic nanoparticles’ size ranges from 1 to 100 nm, and they can couple proteins, medicines, antibodies and other biomedical particles (Li et al., 2022). Combining nano‐metal with drugs can reduce the negative effects of drugs on the immune system. Some nano‐metal particles have antimicrobial and anthelmintic properties, which can improve treatment outcomes alone or with other medicines (Reddy et al., 2020). The primary biomedicine use of these nanoparticles is bioimaging, biosensing, hyperthermy, gene transfer, drug delivery systems and cell labelling (Manjula et al., 2022). The most commonly used metallic nanoparticles for drug delivery are silver, gold, iron oxide, copper and zinc (Chouhan & Mandal, 2021). These nanoparticles in veterinary science have been chiefly used as antiviral and antimicrobial agents (Liew et al., 2022).

### 2.5 Micellar nanostructures

Micelles are drug carriers that consist of amphiphilic polymers and have two parts, the internal part (nucleus) constitutes a hydrophobic sector, and the external part (crown) consists of a hydrophilic sector that has the size of (10–100 nm) (Sanabria, 2021). The particle size can be controlled in different ways, achieving methodology, the aggregates number, the polymers molecular weight and the number of polymers (Li & Zhang, 2021). Other kinds can make polymeric micellar nanoparticles, which divide into four categories by the division of the hydrophilic part. There are four classes of micelles. The first class is phospholipidic micelles, which have phospholipids in their crown. The second class is pluronic micelles, the third class is poly‐l‐amino acid micelles and the fourth class is polyester micellar nanoparticles, which are biocompatible (Carvalho et al., 2020).

### 2.6 Nanoemulsion

**FIGURE 1:**
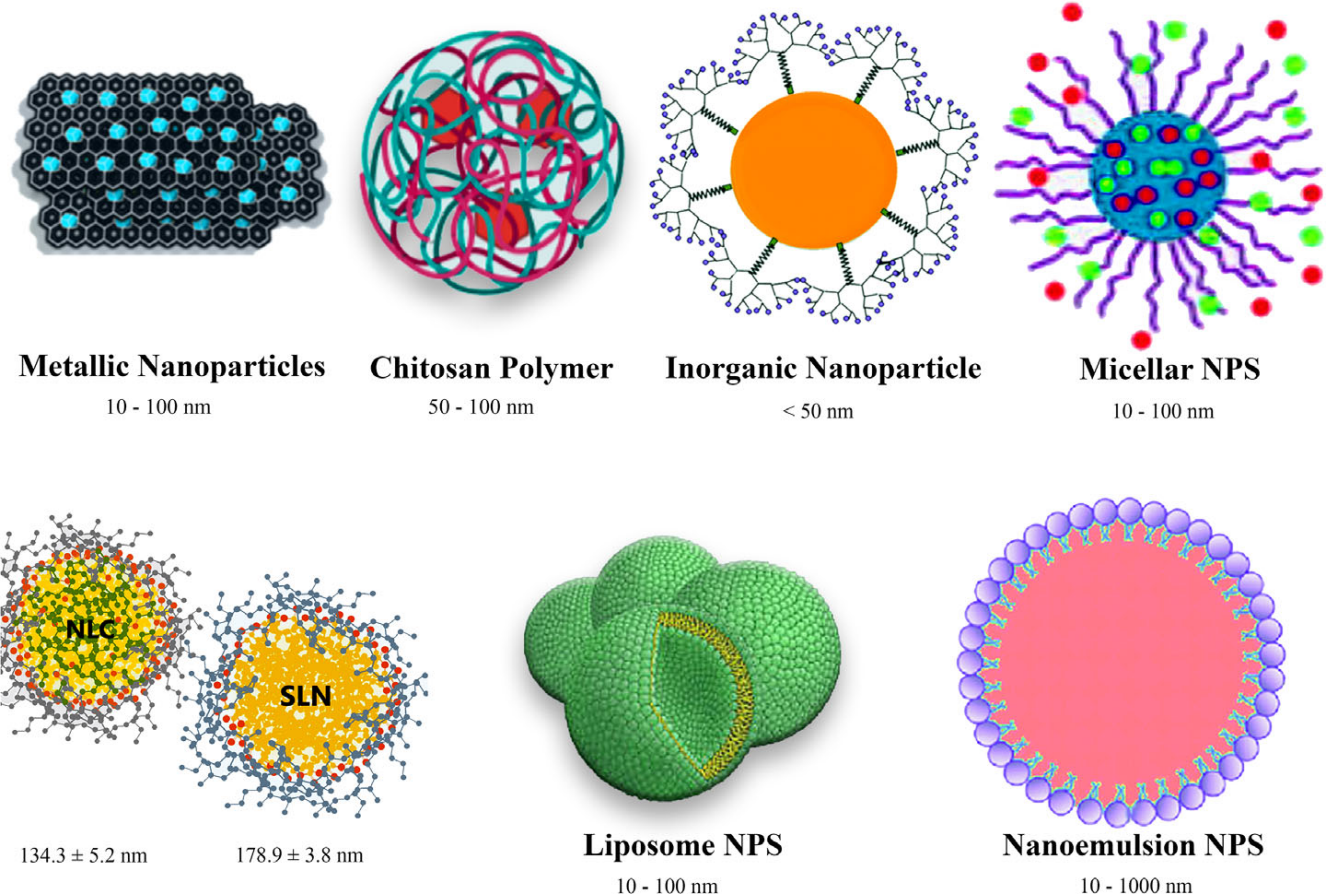
Current applications used in the veterinary medicine field for nanovaccine development.

Nanoemulsions (NEs) are formed from the colloidal dispersion of droplets, developed in at least three parts: water, oil and the emulsifier, and the size might differ (50–1000 nm). It is a kinetically steady and thermodynamically irregular method. Some research has shown the benefit of NE as a nano‐delivery approach for veterinary purposes (Manocha et al., 2022). NE is applicable to transferring medicines into the liquid portions of breeding living organisms or steadily releasing poorly injectable hydrophilic medications (Costa et al., 2021). Nanoemulsions can help improve the bioactivity and steadiness of antiparasitic drugs for treating animal parasitic diseases (Rehman et al., 2020) (Figure 1).

## 3 WHY NANO‐VACCINOLOGY?

In recent years, nano‐vaccinology has developed quickly, leading to the production of ‘Nano‐Vaccines’. Numerous peptides and proteins have recently been introduced as a new treatment method (Askarizadeh et al., 2020). Some of their positive features were: controlled drug release, increased drug stability and improved targeting capacity (Jazayeri et al., 2021). Antigen‐carrying nanoparticles can influence the immune system and improve the T‐cell cytotoxic response against antigens linked to nanoparticle (Huang et al., 2019). This occurs due to the biological potential of several antigen‐expressing cells, which can strongly attract foreign particles like bacteria and microparticles (Cordeiro & Alonso, 2016). Another advantage is regarding lymphoid tissues, the nanoscale size of the particle and proper antigen production and diagnosis (Prasanna et al., 2021). Regarding nanovaccines, the nanoparticles interact with the immunogen in one of three ways: absorption, conjugation or encapsulation (Shen et al., 2018). This cellular nutrition procedure detects antigens to express foreign antigens to other cells in the immune system. Nano‐metric particles can improve the efficacy of vaccines due to their biomedical uses (Du & Sun, 2020). Nanoparticles also have various characteristics as adjuvants for vaccines. However, these materials have some problems, like immunity toxicity and unspecific absorption by the intracranial system (Yun & Cho, 2020). Nanotechnology presents opportunities to use molecules for scientific purposes, where traditional strategies are limited.

## 4 NANOVACCINES INTERVENTION IN VETERINARY MEDICINE SCIENCE AND ANALYSING CURRENT STUDIES

**FIGURE 2:**
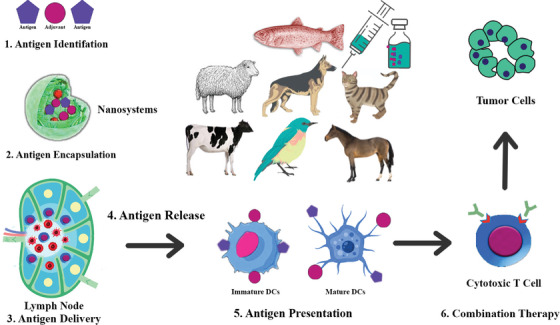
Nanovaccine mechanism of action.

Nanoparticles have received much attention recently because they use a release system or immune system enhancers (Rosales‐Mendoza & González‐Ortega, 2019). In addition, they have emerged as a novel method for vaccination. Moreover, nanovaccines are more efficient than conventional vaccines and trigger blood and cell immune reactions (Loera‐Muro & Angulo, 2018). Nanovaccines inhibit infection by killing infectious agents controlling the immune system. Furthermore, vaccines significantly benefit from nanoparticles formulations, enhancing antigen perception, targeted APCs administration, immunogenicity and slow release of antigens (Sun et al., 2018). Because of their biodegradability, bioavailability and minimal toxicity, most vaccine models containing NPs can be safe and effective alternatives to traditional vaccine formulas (Seyfoori et al., 2021). With the help of nanoparticles as adjuvants or vectors, a new generation of vaccines is produced. For instance, they can efficiently activate the immune response due to the similar size of pathogens and nanoparticles (Xiang et al., 2006). The stimulation of each humoral and cellular immune response followed the application of this kind of vaccine. The advantages of nanovaccines are their best stability in the bloodstream, improved immune system activation, cold chain and no boosters (Prasad, Ghosh, et al., 2021). Using nanoparticles in vaccinology presents two important benefits (Shah et al., 2014). The first benefit is that NPs act as adjuvants and antigens, which increase the antigenicity of conjugated and adsorbed antigens. In this case, they can imitate the characteristics of pathogens like viruses (Dmour et al., 2022). Second, the results of inflammatory cytokines mediate many immune responses activated by nanoparticles. Consequently, different nanoparticle frames are currently advanced for various applications (Chen et al., 2017). These structures can be artificially engineered or found in nature. The engineered particles can mainly be created to select the immune system or avoid interactions. This benefit is linked to the nano‐size (which stimulates absorption by phagocytic cells), mucosa‐associated lymphoid tissues and effective presentation and recognition of antigens (Vinay et al., 2018). The various types of NPs, including virus‐like particles, liposomes, inorganic particles and polymeric particles, have become widely recognized in medical science, particularly in the field of vaccinology (Lei et al., 2020). The emulsion ability of nanoparticles helps synthetic vaccines to enhance immunity and to perform as a vaccine delivery system for different materials. It can cause pliable natural and immune responses. They are used as antigen carriers to improve antigen processing because of their functionality and surface space. These qualities lead to controlled antigen release and effective cell targeting. Nanoparticles have the potential to both increase the half‐life of most vaccines and release antigens in a controlled way (Azadi et al., 2020). Additionally, they can act individually as immune enhancers. However, mixed with immune enhancers, classical adjuvants have more sophistication than improved immunogenicities (Table 1) (Figure 2).

**TABLE 1:** Current status of nano‐vaccinology in veterinary medicine science.

Target	Nanoparticle carrier	Model of study	Result	References
*Anaplasma marginale*	Silica vesicles: thin wall of 6 nm and pore size of 5.8 nm	Mouse	Activate both B‐ and T‐cell immunity	Zhao et al. (2016)
*Leishmania major*	TSA recombinant plasmid and poly(methyl methacrylate) nanoparticles	Mouse	Inducing specific antibody responses	Zarrati et al. (2014)
*L. major*	PLGA nanoparticle	Mouse	Significant induction of nitric oxide production by peritoneal macrophages and increase in splenocyte IFN‐γ production showed the protective effect of PLGA‐CPA/CPB vaccination. The conjugation of the antigen with the PLGA can activate immune responses against *L. major*	Noormehr et al. (2018)
*Echinococcus granulosus*	Polymeric nanoparticles	Dog	Higher cell internalization in murine macrophages and dendritic cells as well as a higher penetration into Caco‐2 cells in vitro	
*Haemonchus contortus*	Poly(lactic‐*co*‐glycolic acid) (PLGA) nanoparticle	Goat	The level of IL‐17 and TGF‐β increased and the faecal eggs and the abomasal worm burdens was reduced	Wang et al. (2021)
*Theileria parva*	Silica vesicle (SV)‐p67C	Cattle	Primed strong Ab and T‐cell responses, stimulation of both high Ab titres and CD4 T‐cell response to p67C	Lacasta et al. (2021)
*Rhipicephalus microplus*	Silica vesicles (SVs) Rhodamine‐labelled SV‐140‐C18 (Rho‐SV‐140‐C18) vesicles	Mouse	Greater accumulation of SVs at the site of injection. The Bm86 antigen biodistribution was traced in lymph nodes, kidney, and liver, this delivery platform successfully elicits antibody responses	Mody et al. (2021)
*Bacillus anthracis*	Cyclic di‐GMP (CDG), polyanhydride nanoparticle	Mouse	Elicited rapid, high titre and immunization that persisted for at least 108 DPI	Kelly et al. (2021)
*B. anthracis*	Amphiphilic polyanhydride nanoparticles	Mouse	Provide controlled antigen delivery, antigen stability, immune modulation and protection in a single dose against a pathogenic challenge and also can stabilize and release functional protective antigen	Petersen et al. (2012)
*Brucella*	PLGA nanoparticles (LPS‐PLGA and OPS‐PLGA)	Mouse	Immune responses have increased on immunoglobulin production. Immune systems were able to remove more bacteria from the body. So improve the immunization process	Afshari et al. (2020)
*Escherichia coli*	Chitosan (CS) and ascorbate chitosan (AsCS) nanoparticles	Broiler chickens	Improving vaccine efficacy via the induction of strong immunity	Mohammed et al. (2021)
*Clostridium perfringens type D*	Chitosan nanoparticle	Mouse	IgA antibody serum level increased sufficiently and IgG antibody titres increased	Poorhassan et al. (2021)
*Leptospira interrogans*	Halloysite clay nanotubes (HNTs) and amine‐functionalised multi‐walled carbon nanotubes (NH2‐MWCNTs)	Golden Syrian hamsters (*Mesocricetus auratus*)	Transfecting CHO cells, inducing IgG immune response	Oliveira et al. (2020)
*Salmonella enteritidis*	Outer membrane proteins (OMP) and flagellin (FLA) of SE [CS‐NP (OMP + FLA)]	Broiler chickens	Antigen‐specific splenocyte proliferation, mucosal and systemic antibody response and the frequency of IFN‐γ‐producing T cells were increased; mRNA levels of toll‐like receptor (TLR) 2 and TLR 4, and cytokines IL‐4 and IL‐10 were up‐regulated	Han et al. (2020)
*Salmonella*	Outer membrane proteins (OMPs) and flagellin (F) protein loaded and F‐protein surface coated chitosan nanoparticles (CS NPs) (OMPs‐F‐CS NPs)	Chicken	Enhanced the specific systemic IgG and mucosal IgA antibody responses as well as reduced the challenge Salmonella load in the intestines	Renu, Han et al. (2020)
*S. enteritidis*	Chitosan‐nanoparticle	Broiler chickens	Induce an antigen‐specific immune response against SE and decrease SE caecal load in broilers	Acevedo‐Villanueva et al. (2021)
*Salmonella*	Outer membrane proteins (OMPs) and flagellin (F) protein (OMPs‐F‐CS NPs)	Layer chickens	Increased the expression of toll‐like receptor in chicken caecal tonsils. (Targets immune cells of chickens and induced antigen‐specific B‐ and T‐cell responses)	Renu, Markazi et al. (2020)
*Mycobacterium tuberculosis*	Polymeric nanocapsules (NCs) with an oily core and a polymer shell made of chitosan (CS) or inulin/polyarginine (INU/pArg)	Cell lines?	INU:pArg:Ag NCs induced an adequate immunoglobulin A (IgA) response	Diego‐González et al. (2020)
*Pseudomonas aeruginosa*	Poly lactic‐*co*‐glycolic acid	Rabbit	Effective in immunization	Safari Zanjani et al. (2018)
*Brucella melitensis*	Poly(lactic‐*co*‐glycolic acid) (PLGA)	Mouse	Increase in the total IgG and IgM antibody titres and high level of protection	Maleki et al. (2019)
*Flavobacterium columnare*	Mucoadhesive polymer chitosan‐complexed nanovaccine (CS‐NE)	Red tilapia (*Oreochromis* sp.)	Better adsorption onto the mucosal surfaces, elicited great vaccine efficacy and modulated the MALT immune response. Provided effective delivery system for the induction of a mucosal immune response against columnaris disease in tilapia	Kitiyodom et al. (2021)
*Staphylococcus aureus*	Mesoporous silica nanoparticles (MSN)	Mouse	Improved CD8+ T‐cell responses while maintaining CD4+ T‐cell responses and humoral immunity. Prevented and treated superficial infection and decreased bacterial invasiveness	Chen et al. (2020)
*Yersinia pestis*	Polyanhydride nanoparticle	Mouse	Nanovaccine delivery system enables induction of both rapid and long‐lived protective immunity against *Y. pestis*	Wagner et al. (2019)
Avian pathogenic *E. coli* (APEC)	Nano‐sized proteolipids enriched with various immunogenic molecules	Broiler chicken	Involvement of both non‐specific immune responses and specific antibody and cytokine responses in the APEC_OMV‐mediated protection	Hu et al. (2020)
*Mycobacterium avium* subsp. *paratuberculosis*	Polyanhydride nanoparticles (PAN)	Mouse	Increase in the levels of antigen‐specific T‐cell responses. High percentages of triple cytokine (IFN‐γ, IL‐2, TNF‐α) producing CD8+ T cells. Significant levels of double (IFN‐γ, TNF‐α) and single cytokine (IFN‐γ) secreting CD8+ T cells	Thukral et al. (2020)
BRSV	Polyanhydride nanoparticles (CPTEG:CPH nanoparticles)	Neonatal calf	Reduced pathology in the lungs, reduced viral burden and decreased virus shedding	McGill et al. (2018)
*Swine influenza virus*	Biodegradable polyanhydride nanoparticles	Mouse	Augmented antigen‐specific cellular immune response in pigs, with promise to induce cross‐protective immunity	Dhakal et al. (2017)
*Avian influenza virus*	Polymer (2‐hydroxyethyl methacrylate)	Hy‐Line chickens	Reduction in virus shedding from the oropharynx	Shan et al. (2010)
*Infectious bursal disease virus*	Unilamellar liposome	Broiler chickens	Elevated humoral antibody titre of IgG, cellular immunity (INF‐γ levels) increased and induce highly protective level of immune response	
*Rhabdovirus*	Targeted carbon nanotubes	Fish	Enhanced uptake by carp macrophages and immune‐related tissues, which would then trigger strong immune responses against spring viremia of carp virus (SVCV) infection	Zhang et al. (2020)
*Zika virus*	Nanoparticles made with a recombinant form of *Z. virus* nonstructural protein (NP‐∆NS1)	Mouse	Boost antibody production and increase the efficiency of subunit vaccines	Favaro et al. (2021)
*Classical swine fever virus* (CSFV)	Ferritin nanoplatform	Mouse	High ferritin‐assisted humoral and cellular immunities	Zhao et al. (2021)
*Bovine viral diarrhoea virus* (BVDV)	Silica nanoparticles	Mouse	Higher antibody response and higher cell‐mediated response	Mody et al. (2014)
*Bovine viral diarrhoea virus 1* (BVDV‐1)	Hollow type mesoporous silica nanoparticles with surface amino functionalisation (HMSA)	Sheep	The long‐term cell‐mediated immune responses were detectable up to 4 months. The cell‐mediated immune responses were consistently high. Gives balanced immune responses in a production animal	Mahony et al. (2015)
*Newcastle disease virus*	Chitosan nanoparticles containing the lentogenic live virus vaccine against NDV (NDV‐CS‐NPs)	Chicken	Induced better protection of immunized specific pathogen free chickens	Zhao et al. (2012)
Foot‐and‐mouth disease virus	Gold nanoparticles	Guinea pigs	The titre and sensitivity of the raised antibodies were maximal. Antibody biosynthesis was accompanied by increased production of pro‐inflammatory cytokines (especially IFN‐γ) and by stimulation of the respiratory activity of peritoneal macrophages	Dykman et al. (2015)
Porcine reproductive and respiratory syndrome virus (PRRSV)	PLGA [poly(lactide‐*co*‐glycolides)] nanoparticles	Pigs	Reduced the lung pathology and viremia, and the viral load in the lungs. Immunologically, enhanced innate and adaptive immune cell population and associated cytokines with decreased secretion of immunosuppressive mediators were observed at both mucosal sites and blood	Dwivedi et al. (2012)
Ovarian cancer	Poly(lactic‐*co*‐glycolic acid) (PLGA)	Cell lines	Delaying the growth and inhibiting the metastasis of OC	Zhang et al. (2022)
Cancer	Poly(d, l‐lactide‐*co*‐glycolide) (PLGA) nanoparticles	Mouse	Increase antigen cells uptake, increase efficiency to delay tumour development	Yang et al. (2018)
Cancer	(InAc‐NPs) using inulin acetate (InAc)	Mouse	Produce storing serum antibody titres against encapsulated antigen. Experimentally, increase cytokines. Complete protection from metastasis of intravenously injected melanoma cells to lungs	Rajput et al. (2018)

## 5 CHALLENGES IN THE NANOVACCINES MANUFACTURING PROCESS, QUALITY CONTROL AND REGISTRATION

Considering the cost‐control processes when designing nanovaccines is necessary since NPs production is valuable compared with typical drugs (Pinheiro et al., 2011). The primary challenge in mass‐producing nanovaccines is the high cost of scaling up production and the production process itself. Analysing methods, large‐scale production, in‐process quality control and final product testing all require a significant investment of resources, both in terms of time and money (Usman et al., 2020). Outsourcing the nanovaccine manufacturing line is another way to defeat the barrier, as numerous companies worldwide specialize in various nanoparticles production (Ferrari, 2005). Another possible alternative is collaboration with research and academic organizations. Therefore, the manufacturing process of nanovaccines must reveal adequate strength and consistent quality in mass production (Ahmad et al., 2020). Another challenge is the lack of harmony in quality control tests and manufacturing. For example, there are no standard methods in the pharmaceutical industry for in‐process quality control and the end products because innovative nano‐drugs are typically complex and challenging to separate (Dacoba et al., 2020). Nano‐drugs’ size is defined by the critical quality attribute (CQA). Some factors, such as charge, surface coating, morphology and shape, are other CQA to nano‐drugs (Rawal et al., 2019). Even minor variations in particle size or large size dispersal in the scale might change the product's bioavailability. Furthermore, the small size creates particular conditions for the analytical methods (Fifis et al., 2004). In addition, particle shape can affect the kinetics of cell uptake, the mechanisms of uptake, the level of uptake and intracellular distribution; therefore, toxicity can be changed (Clogston et al., 2020). For instance, the shape of (Au) impacts the nanoparticle cellular internalization (Rodríguez‐León et al., 2019; Salatin et al., 2015; Spadavecchia et al., 2016). In one study, the cellular uptake was most significant for triangle‐shaped particles, and for star‐shaped ones was lowest (Jiang et al., 2012). The surface layer also influences cell capture nanoparticles. For example, phagocytic cells catch nanoparticles with a negative charge faster than neutrals and are removed more quickly than the latter (Djemaa et al., 2018; Sercombe et al., 2015). Meanwhile, nanoparticles with a positive charge can link with negatively charged proteins and stay longer in the blood (Djemaa et al., 2019; Park et al., 2016). One of the major challenges is predicting the relationship between in vivo and in vitro manners of nanoparticles. The main elements that need to be studied using in vivo samples are tissue accumulation, cellular interactions, transportation and biocompatibility (Pokrajac et al., 2021). The study of the accumulation and the long‐time release profile must be idealized due to the persistence of nanoparticles in the blood or their removal in tissues and the lack of knowledge of long‐term impacts (Lowry et al., 2019).

## 6 NEW DEVELOPMENTS FOR A BRIGHTER FUTURE

**FIGURE 3:**
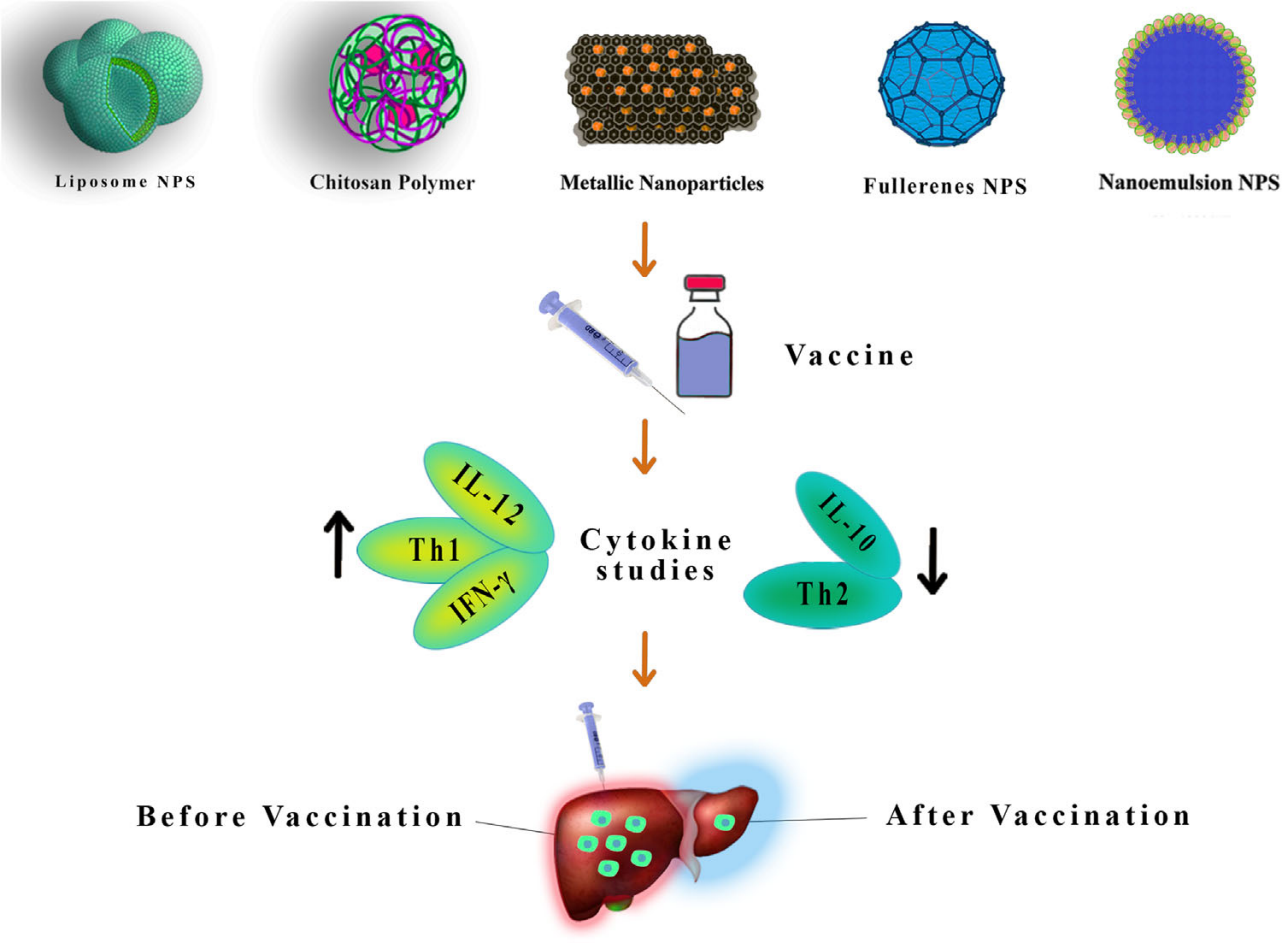
T‐cell immunity against different pathogens.

Vaccination includes a series of microbiology, immunology, molecular biology, dose application rules, production cost and return on investment (Qin et al., 2021). Creating a production against infections for human and animal immune systems is the highest purpose of any novel vaccination method. Despite having a significant influence on humans, vaccination for veterinary is also concerned with great production and animals’ health. Nanoscience assists with veterinary parasite vaccination, enhancing their potency. Vaccines that use nanoparticles as adjuvants have more power to stimulate both cellular immune reactions and antibody responses (Singh, 2021). In addition, nanotechnology can improve the activation of the immune system by creating greater antibody protection and concentration. This activation occurs by simultaneously triggering the MHC class I and II routes (major histocompatibility complex), or improving the antigen lifetime and their visibility, to reach the host immunity (Beg et al., 2020; Wang et al., 2018). One of the best methods to raise the efficacy of unique and eradicated vaccines is to use unique carriers, including living vectors, micro‐ or nanoparticles and plasmid DNA (Jia et al., 2018; Zhang et al., 2019). The crucial part of natural immunity and the responses of vaccine aids are important points in vaccine production that are often ignored. Understanding the T‐cell immune response is crucial, and studying pathogen‐specific T‐cell immunity could provide valuable insights and signatures to aid in the development of vaccines against infectious diseases (Fajardo et al., 2022). Specific mechanisms for immune signalling must be confirmed for reprogramming of immune cascade. Vaccines are designed innovatively to reach complete protection. Antibody‐dependent enhancement can hinder vaccine development by worsening the immune response. Therefore, it is important to strive for a balanced introduction of neutralizing antibodies, T‐cell immunity against various pathogens and antibody effector functions to optimize vaccine efficacy (Najafi‐Hajivar et al., 2016) (Figure 3). The use of nanoparticles in veterinary vaccines is less restricted compared to human vaccines. This means that there is more flexibility in choosing from a variety of particles with different formulas for veterinary vaccination. In contrast, only a limited number of vaccine particles are currently used for human vaccines.

## 7 CONCLUSION

‘Nano‐Vaccinology’ is the science of nano‐sized materials with great capacity. The clinical and laboratory scale can expand beyond the boundaries by a more immunogenic and stabilized release against non‐infectious and infectious diseases. In the storage and synthesis of nanovaccines, quality is a concern in nanovaccines commerce regarding desirable surface effects. For efficiently preventing non‐infectious pathogenic and cancerous diseases in immune‐tolerant patients, the nanovaccines have opened an entrance to boundless hopes. More studies focusing on partnerships with commercial companies lead to the rapid commercializing of nanovaccines.

## AUTHOR CONTRIBUTIONS

*Conceptualization; supervision*: Hassan Borji. *Methodology; formal analysis and investigation; writing – original draft preparation; writing – review and editing*: Soheil Sadr. *Formal analysis and investigation; writing – original draft preparation; writing – review and editing*: Parian Poorjafari Jafroodi. *Writing – original draft preparation*: Mohammad Javad Haratizadeh and Zahra Ghasemi. *Funding acquisition*: No Funding. *Resources*: No Resources. *Figure Producer*: Ashkan Hajjafari.

## CONFLICT OF INTEREST STATEMENT

The authors declare no conflicts of interest.

### FUNDING INFORMATION

No funding was received for conducting this study.

### ETHICS STATEMENT

All applicable international, national and/or institutional guidelines for the care and use of animals were followed.

### PEER REVIEW

The peer review history for this article is available at https://publons.com/publon/10.1002/vms3.1221.

## Data Availability

The data sets generated during and/or analysed during the current study are available from the corresponding author on reasonable request.
